# Breakdown of Phylogenetic Signal: A Survey of Microsatellite Densities in 454 Shotgun Sequences from 154 Non Model Eukaryote Species

**DOI:** 10.1371/journal.pone.0040861

**Published:** 2012-07-16

**Authors:** Emese Meglécz, Gabriel Nève, Ed Biffin, Michael G. Gardner

**Affiliations:** 1 IMBE UMR 7263 CNRS IRD, Aix-Marseille University, Marseille, France; 2 School of Biological Sciences, Flinders University, Adelaide, Australia; 3 Australian Centre for Evolutionary Biology and Biodiversity, School of Earth and Environmental Science, University of Adelaide, Adelaide, Australia; 4 Evolutionary Biology Unit, South Australian Museum, Adelaide, Australia; University of Poitiers, France

## Abstract

Microsatellites are ubiquitous in Eukaryotic genomes. A more complete understanding of their origin and spread can be gained from a comparison of their distribution within a phylogenetic context. Although information for model species is accumulating rapidly, it is insufficient due to a lack of species depth, thus intragroup variation is necessarily ignored. As such, apparent differences between groups may be overinflated and generalizations cannot be inferred until an analysis of the variation that exists within groups has been conducted. In this study, we examined microsatellite coverage and motif patterns from 454 shotgun sequences of 154 Eukaryote species from eight distantly related phyla (Cnidaria, Arthropoda, Onychophora, Bryozoa, Mollusca, Echinodermata, Chordata and Streptophyta) to test if a consistent phylogenetic pattern emerges from the microsatellite composition of these species. It is clear from our results that data from model species provide incomplete information regarding the existing microsatellite variability within the Eukaryotes. A very strong heterogeneity of microsatellite composition was found within most phyla, classes and even orders. Autocorrelation analyses indicated that while microsatellite contents of species within clades more recent than 200 Mya tend to be similar, the autocorrelation breaks down and becomes negative or non-significant with increasing divergence time. Therefore, the age of the taxon seems to be a primary factor in degrading the phylogenetic pattern present among related groups. The most recent classes or orders of Chordates still retain the pattern of their common ancestor. However, within older groups, such as classes of Arthropods, the phylogenetic pattern has been scrambled by the long independent evolution of the lineages.

## Introduction

Eukaryote genomes contain vast numbers of tandemly repeated DNA motifs of 1–6 base pairs. As widely used molecular markers, microsatellites have their strength in their high variability [1]. The relative power of the microsatellites over Single Nucleotide Polymorphisms (SNPs) due to the high variability of microsatellites is 4–12 fold for population genetic structure [2], [3], 5–12 fold for association or linkage disequilibrium studies [4] and 10 fold for sibling reconstruction [5]. The application of high throughput next generation sequencing (NGS), is amenable both to SNP and microsatellite development and it appears that these molecular markers will both be widely used for some time to come.

The origin and spread of microsatellites within a genome is a puzzling question [6]. A more thorough understanding of factors influencing the genomic distribution of microsatellites would facilitate their continued use as a molecular marker and contribute to a general understanding of microsatellite evolution in genomes. Microsatellite formation cannot be explained by chance alone, since the expected density of microsatellites, assuming random association of DNA bases, is far lower than their observed genome wide density [7]. There are two major mutually non-exclusive hypotheses for microsatellite formation: *de novo* formation of microsatellites from unique sequences by point mutations [8], [9] and spread of microsatellites into new locations by transposable elements [10]. Following the formation of proto-microsatellites, their expansion is thought to be primarily due to replication slippage [7], [11] and a slightly inefficient mismatch repair system [12]. Polymerase slippage rate increases with the number of repeat units and is inversely correlated with repeat unit length [13]. Several studies have demonstrated positive correlation between mutation rate and allele size [14]–[16] although contractions become more likely than expansions with increasing number of repeats [17]. As a consequence, mutation rate of microsatellites varies across loci, alleles and among species [18]. It is therefore difficult to determine what the key factors are that influence microsatellite distribution in different species. Slippage mechanism, mismatch repair, transposable element types and their abundance are all factors that can differ between phylogenetic groups, thus resulting in variable microsatellite coverage and composition (*i.e.* proportion of different motif types). The differences have even been suggested to follow a consistent pattern such that microsatellite content may be used as a phylogenetic signal [19].

Several studies describe microsatellite density or coverage at a genome scale, but these are often hampered by a limited taxonomic range (e.g. Nematodes [20], insects [21], [22], fungi [23], plants [24], Tritryps [25]) or a reliance on model species with complete genomes [19], [26]–[30]. An important exception is the paper by Tóth *et al*. [31], where the authors used sequences from 3764 species including plants, fungi and animals. However, since these sequences came from GenBank, the whole dataset is very strongly biased towards 14 model species that represent the large majority of the data. Furthermore, species were grouped into arbitrary units such as ‘mammals other than rodents or primates’, or ‘vertebrates other than mammals’, which prevented a comparison between monophyletic lineages and thus within group variability could not be estimated. Although the authors took special care to decrease a probable database bias towards coding sequences, it is still unlikely that their data can be regarded as representative random sample of all of the investigated genomes. During the twelve years since the publication of Tóth *et al*.’s paper, NGS has become an established tool in obtaining the snapshot of a variety of genomes of non-model organisms providing a relatively unbiased representation of genomes [32]. NGS genome snapshots are thus likely to be a far more accurate random sampling of genomic sequences than GenBank data mining. Furthermore, even species with whole genome sequences are likely to present some bias, since in the vast majority of the cases, the centromeric/telomeric and other regions with repetitive DNA are usually not assembled and their copy number is difficult to establish in the non-positioned scaffolds.

In this study we use 454 shotgun sequences from 154 non-model species of Eukaryotes, to compare microsatellite coverage (defined as the number of bases of microsatellite per Mb of DNA) and microsatellite composition (the proportion of different motif types) between varied taxonomic groups to ascertain to what extent a phylogenetically consistent pattern exists. Both the presence and absence of such a phylogenetic signal are likely to throw light on the evolution of microsatellites. For example, evidence for the maintenance of inherent differences between major evolutionary groups invokes a varied yet functional contribution of these repetitive elements within disparate genomes. Alternatively, inconsistencies within lineages call for a greater role of random processes for explaining microsatellite distributions.

## Results

Although microsatellites are often defined as tandem repetitions of 1–6 bp motifs, [33], in this paper microsatellites are defined as five or greater tandem repetitions of any 2–6 bp motifs. Homopolymer tracks (mononucleotide repetitions) are treated separately; they are thus not included in the total coverage of microsatellites unless stated otherwise. Microsatellite content was measured in two ways: microsatellite coverage is the number of bases of microsatellites per Mb of DNA; and microsatellite composition is the proportion of microsatellite coverage of different motif types.

### Microsatellite Total Coverage

**Figure 1:**
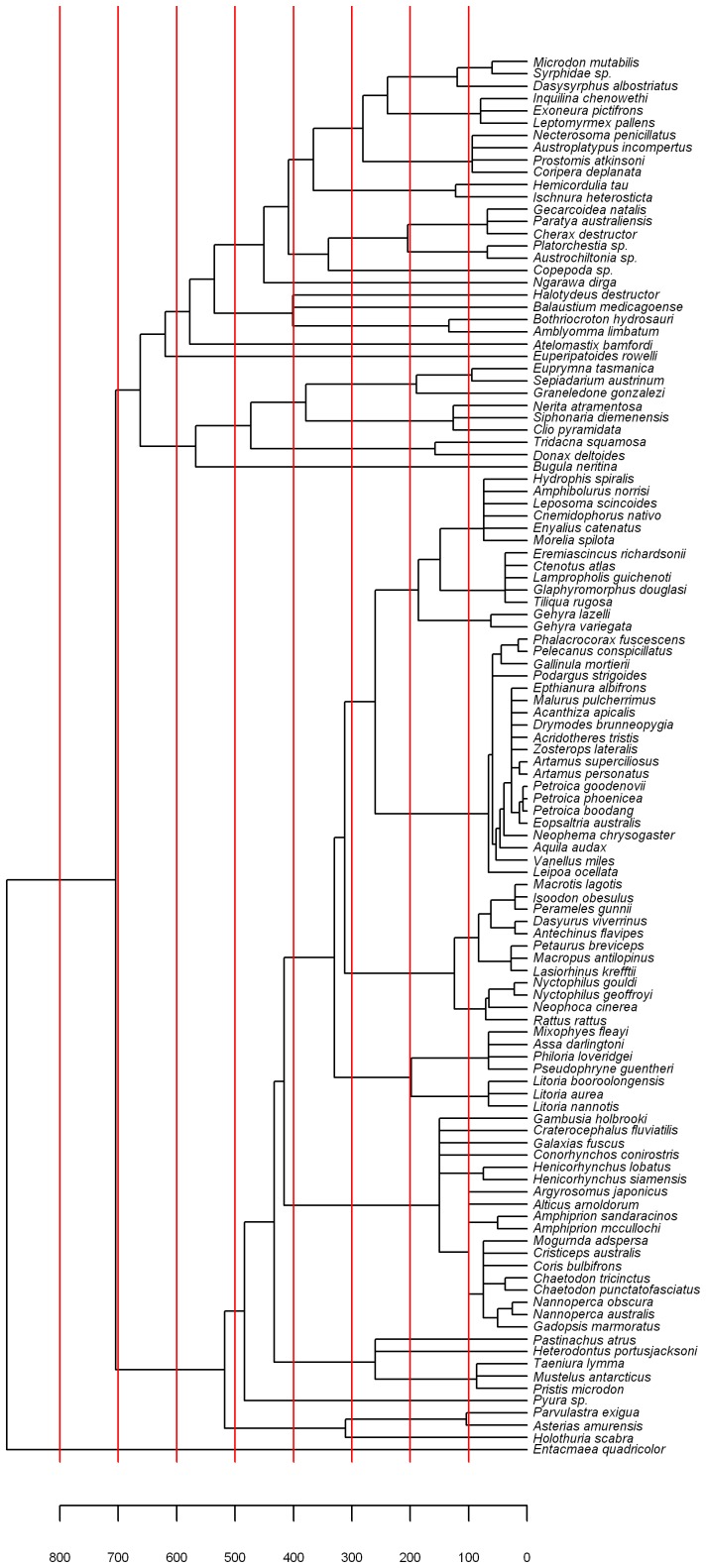
Phylogenetic tree of the animal species studied. The cutoff limits used for autocorrelation analyses of microsatellite coverage are indicated in red.

**Figure 2:**
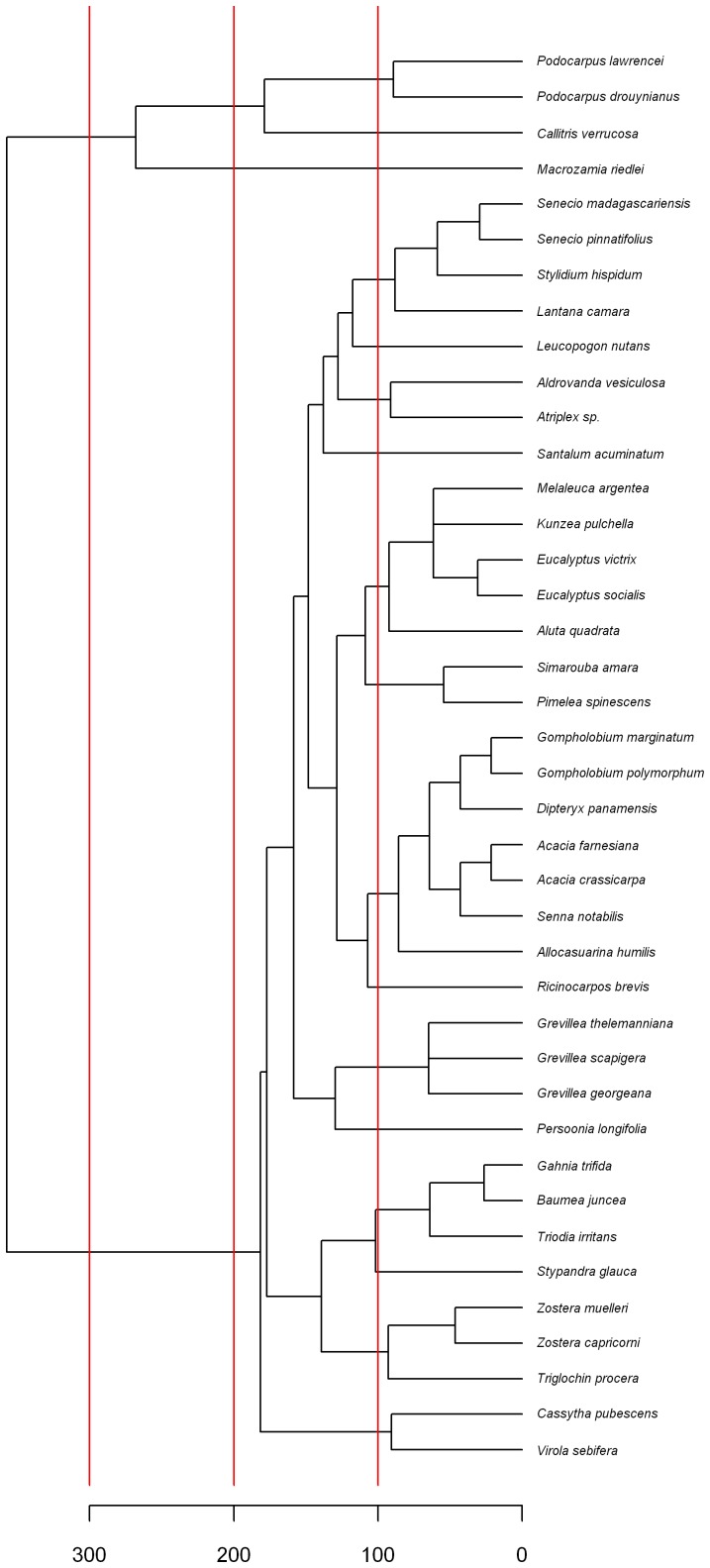
Phylogenetic tree of the plant species studied. The cutoff limits used for autocorrelation analyses of microsatellite coverage are indicated in red.

**Figure 3:**
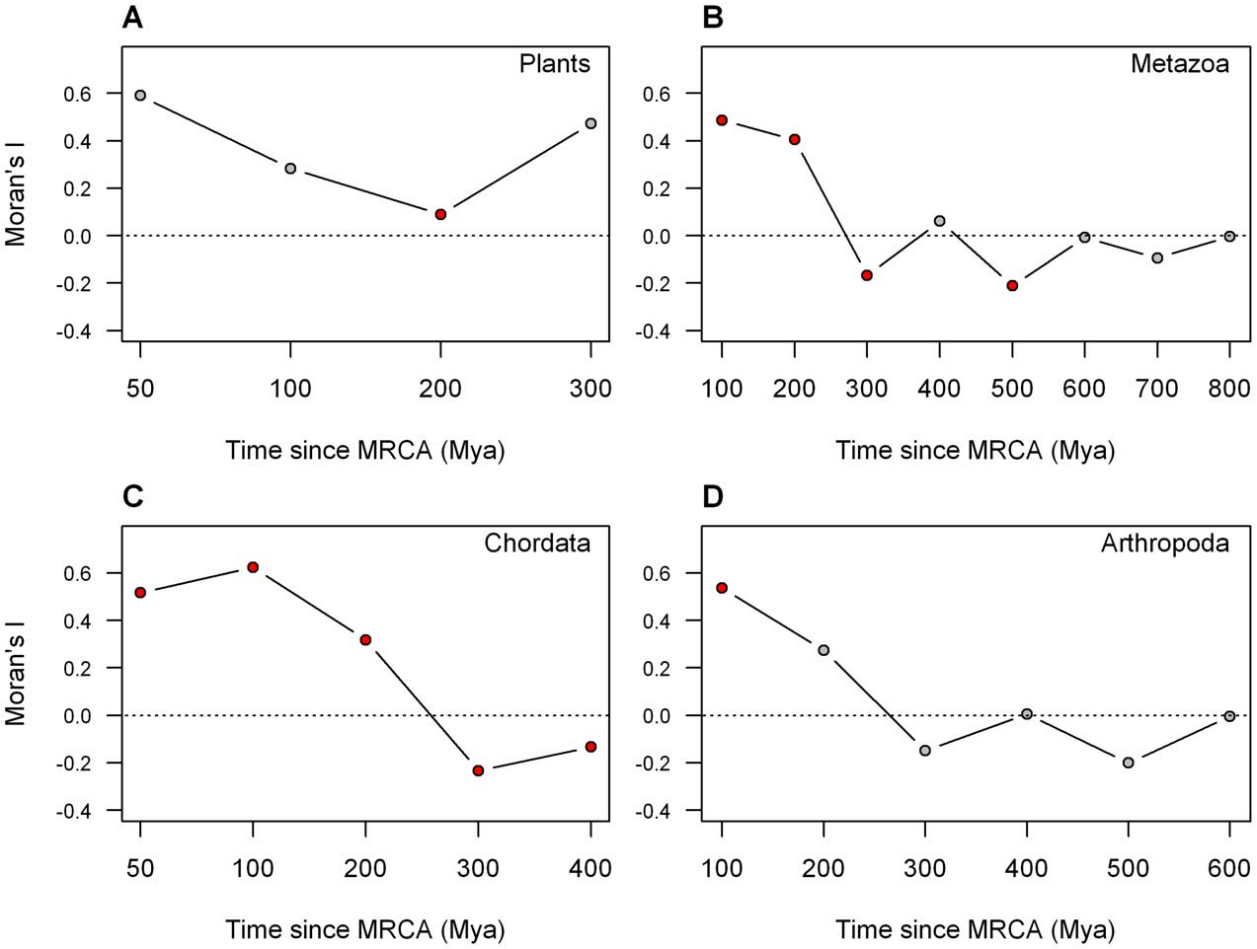
Autocorrelation analyses of total microsatellite coverage. (A) Plants, (B) Animals, (C) Chordates, (D) Arthropods. Red symbols: P<0.05, grey symbols: P≥0.05.

A phylogenetic tree was constructed on the basis of the divergence times between species separately for animals and plants (Figures 1 and 2, respectively) to guide the comparison of microsatellite coverage between clades. Based on these trees, correlogram analyses were conducted at different time scales. For each time limit, the largest possible clades, which had their most recent common ancestor (MRCA) younger than the cut off were identified and their microsatellite coverage were compared. If microsatellite coverage was dependent on divergence time, one would expect a decreasing positive autocorrelation with increasing divergence time. Correlograms were computed for plants (Figure 3A) and animals (Figure 3B) separately. Since the vertebrates were overrepresented within animals (75 species out of 114), a separate correlogram is presented for vertebrates (Figure 3C). Arthropods are also analyzed separately, since this is the animal phyla with the second most species studied (24 species, Figure 3D).

Correlogram analyses indicated positive autocorrelation of the total microsatellite coverage for clades with the most recent common ancestor (MRCA) younger than 200 Mya (Figure 3). Moran’s *I* values for divergence time of 200 Mya or less were positive when analyzing plants, animals, vertebrates or arthropods, but not always significant, probably due to the insufficient number of clades or the small number of species in some clades. However, from 200 Mya upwards, autocorrelation generally decreased with increasing divergence or even became significantly negative. From 600 Mya upwards, the signal was non-significant, when all Metazoa were considered.

**Figure 4:**
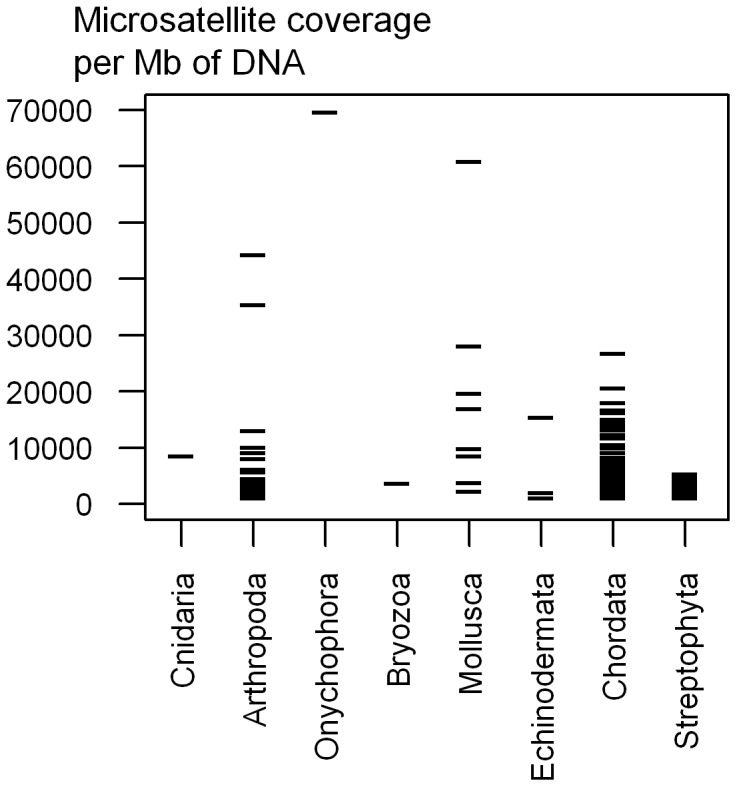
Microsatellite coverage by phyla. Microsatellite coverage is the number of bases of microsatellites (di-hexanucleotide motifs) per Mb of DNA.

**Figure 5:**
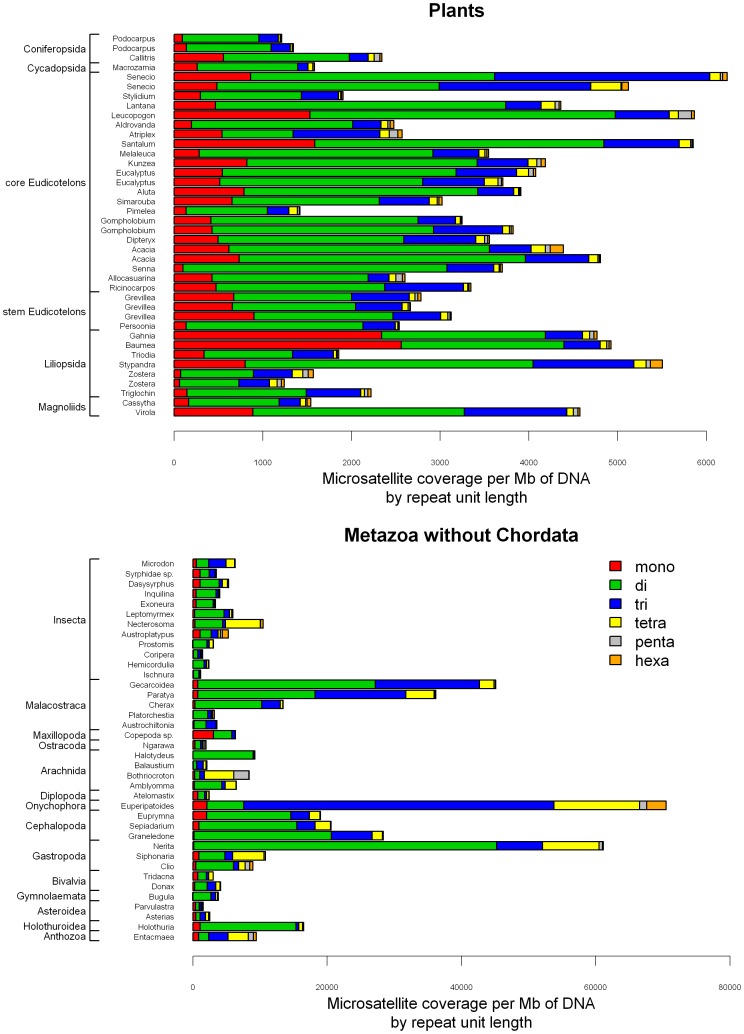
Microsatellite and homopolymer coverage by repeat unit length for plants and Metazoa without Chordata. Microsatellite coverage is the number of bases of microsatellites per Mb of DNA. Coverages of different motifs of the same repeat unit length (mono-hexa) are pooled. Note that the scales of the horizontal axes are different. Species follow the same order as in Dataset S1 and in Figures 1 and 2.

**Figure 6:**
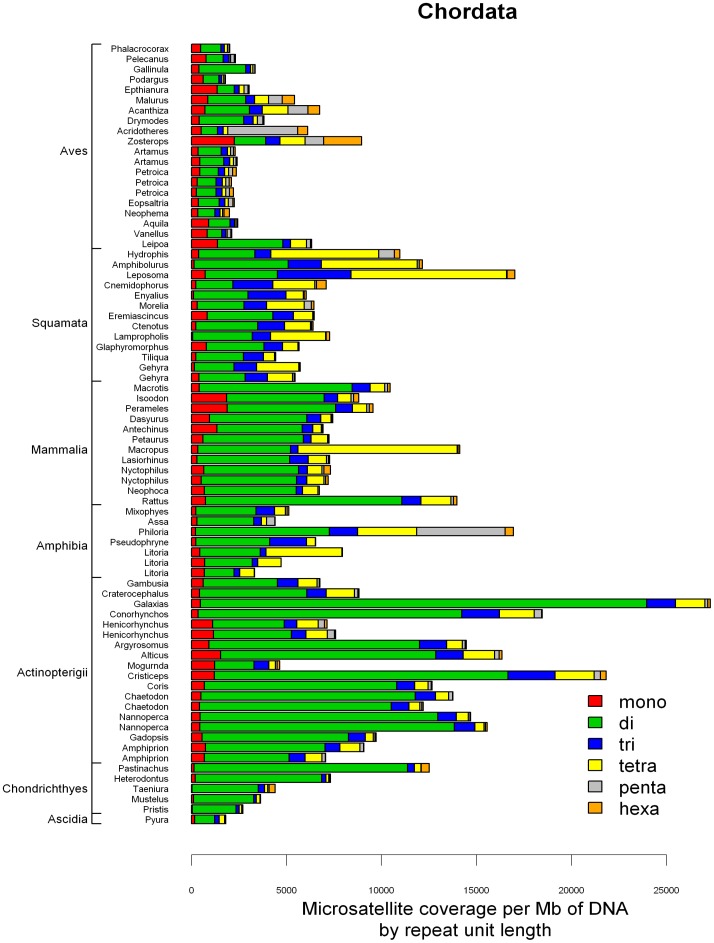
Microsatellite and homopolymer coverage by repeat unit length for Chordata. Microsatellite coverage is the number of bases of microsatellites per Mb of DNA. Coverages of different motifs of the same repeat unit length (mono-hexa) are pooled. Species follow the same order as in Dataset S1 and in Figure 1.

Wilcoxon tests indicated that microsatellite coverage was significantly different between plants and animals when comparing all microsatellites (W = 3644, P = 4E-8) and also separately for di- (W = 3189, P = 1.8E-4), tetra- (W = 4222, P = 1.2E-15), penta- (W = 3769, P = 8.6E-10), and hexanucleotid motifs (W = 3263, P = 5.2E-5). Plants had significantly less microsatellites than animals in general, but there were some exceptions (Figures 4, 5, 6).

For plants, although Moran’s *I* values were positive for all time cut off limits, the autocorrelation was significant only at 200 Mya with a low Moran’s *I* value (0.089). At 200 Mya cut off we are comparing all studied Magnoliophyta (36 species) in one clade to three species of Coniferophyta and one species of Cycadophyta. The results obtained by the autocorrelation is also reflected in Figure 5, where the Conferopsida and Cycadida species appear to have lower microsatellite coverage than the Magnoliophyta (Wilcoxon test: W = 9, P = 0.001). Variabilities of coverage within and between clades of Magnoliophyta are comparable, which is likely to be a consequence of the recent divergence time between the considered clades. It is also important to note that, although the microsatellite coverage varies among plant species, the overall low microsatellite content of plants compared to animals makes this group more homogeneous than the Metazoa clade; the lowest coverage in plants is only about 5 times lower than the highest, while this ratio is around 60 in animals (Figure 5, Dataset S1). Thus the Steptophyta clade is generally characterized by low microsatellite content, and little variability among species compared to the animals.

Comparison of microsatellite coverage of vertebrates revealed a significant positive autocorrelation within clades with MRCA younger than 200 Mya. At this time limit, the clades correspond roughly to vertebrate classes: birds, Squamata, mammals, amphibians, bony fishes. Actinopterigii had significantly more microsatellites than Tetrapods (Figure 6; Wilcoxon test: W = 778, P = 3.2E-5). Within the Tetradopa, the microsatellite coverage of Amphibia was not different from the Amniota (W = 142, P = 0.694), but Squamata had significantly more microsatellites than birds (W = 22, P = 2E-5) and mammals had significantly more microsatellites than birds and Squamata combined (W = 335, P = 2E-4). This last difference was probably due to the low number of microsatellites in birds. However, due to the contingencies of our dataset, this pattern was difficult to depict in the other phyla.

Within Arthropoda the most striking observation is the exceptionally high number of microsatellites of Decapoda, but care should be taken, since this order was represented by only three species (*Paratya australiensis, Gecarcoidea natalis, Cherax destructor*, Figure 4). No other tendency was detected, variability within most of the clades older than 100 Mya was not negligible and the coverage ranges were largely overlapping. The sampling of other phyla does not allow us to do systematic comparisons between clades. However, since many of these phyla are poorly represented in the literature, it is important to present them even if generalization is not possible for these groups. Variability of microsatellite coverage was extremely high among these species (Figures 4, 5). The species with the highest microsatellite coverage of the entire dataset is an Onychophora, but since it is the only species of this phylum in our dataset, we cannot say if it is an outlier, or a middle range representative of the *ca.* 200 species of this phylum. Within Mollusca, microsatellite content is highly variable and especially so within Gastropoda (snails) where a six fold difference was found among the species studied. Both Bivalvia species have low microsatellite coverage, and all three species from Cephalopoda have high coverage, but generalization is difficult due to the low number of species.

### Microsatellite Composition by Repeat Unit Length

We describe microsatellite composition by the proportional coverage of different motif types within microsatellites. In this section, different motifs are pooled by repeat unit length, and the coverage of each repeat unit length is compared to the total microsatellite and homopolymer coverage. Since the cut off limits of microsatellites of different repeat unit length and homopolymers is arbitrary (5 repetition for di-hexa motifs and 12 for homopolymers), the proportions by themselves should not be over interpreted as it would be different with other cut off limits. We use them here for the interspecies comparison, which is valid, since the same criteria are used for each species.

Dinucleotide motifs were the most frequent in the majority of the species (136 out of 154), and often their coverage is higher than the sum of the other motifs (124 species) (Figures 5, 6 and S1). Remarkable exceptions are *Entacmaea quadricolor* (Cnidaria), *Bothriocroton hydrosauri, Balaustium medicagoense* (Arthropoda:Arachnida), *Euperipatoides rowelli* (Onychophora), *Acridotheres tristis, Zosterops lateralis* (Chordata: Aves*), Hydrophis spiralis, Leposoma scincoides, Cnemidophorus nativo* (Chordata: Squamata), where the dinucleotide microsatellite content was 30% or lower of the total coverage. However there was great variability in what was the second most frequent motif class found in each species. While in all plant species studied trinucleotide microsatellites had higher coverage than tetranucleotides, in Chordata tetranucleotides generally outnumbered the trinucleotides, especially within Tetrapods. No clear trends existed among the remaining species (non-Chordate Metazoans). Finally, penta- and hexanucleotide microsatellites were clearly the rarest motif classes with the notable exceptions of *Austroplatypus incompertus* (Arthropoda: Insecta), *Philoria loveridgei* (Chordata: Amphibia), *Malurus pulcherrimus*, *Acridotheres tristis, Zosterops lateralis* (Chordata: Aves), where the sum of penta- and hexanucleotide motifs reached more than 30% of the total microsatellite coverage. Homopolymer coverage relative to microsatellite coverage varied strongly between taxonomic groups. It was generally higher in plants and also in birds than in other animals.

Analyzing both total microsatellite coverage and the proportions of microsatellite coverage by repeat unit length, plants were characterized by a fairly homogeneous distribution. Within generally low microsatellite coverage compared to most animal species, dinucleotide motifs were the most frequent followed by trinucleotide motifs, while tetra-hexa motifs were much rarer. Homopolymer proportions could be relatively high. Birds have similar distributions and coverage, but there were a few species with exceptionally high proportions of tetra-hexanucleotide motifs (*Zosterops lateralis, Acridotheres tristis, Acanthiza apicalis*). Squamates had more microsatellites and less homopolymers than birds, and were characterized by a profile of high tetranucleotide proportions and to a lesser extent trinucleotide proportions were also important. The other Chordata have similar profiles to reptiles but the proportion of dinucleotides was generally higher than in reptiles. For the remaining phyla, however no general pattern emerged. Just as total microsatellite coverage varied within classes and orders, motif length proportions can be markedly different between even closely related species. For example, one species of the Ixodida order, *Amblyomma limbatum,* had primarily dinucleotide microsatellites, while *Bothriocroton hydrosauri* from the same order have extremely low dinucleotide and very high tetra and pentanucleotide coverage.

### Microsatellite Composition by Motif Sequence

**Figure 7:**
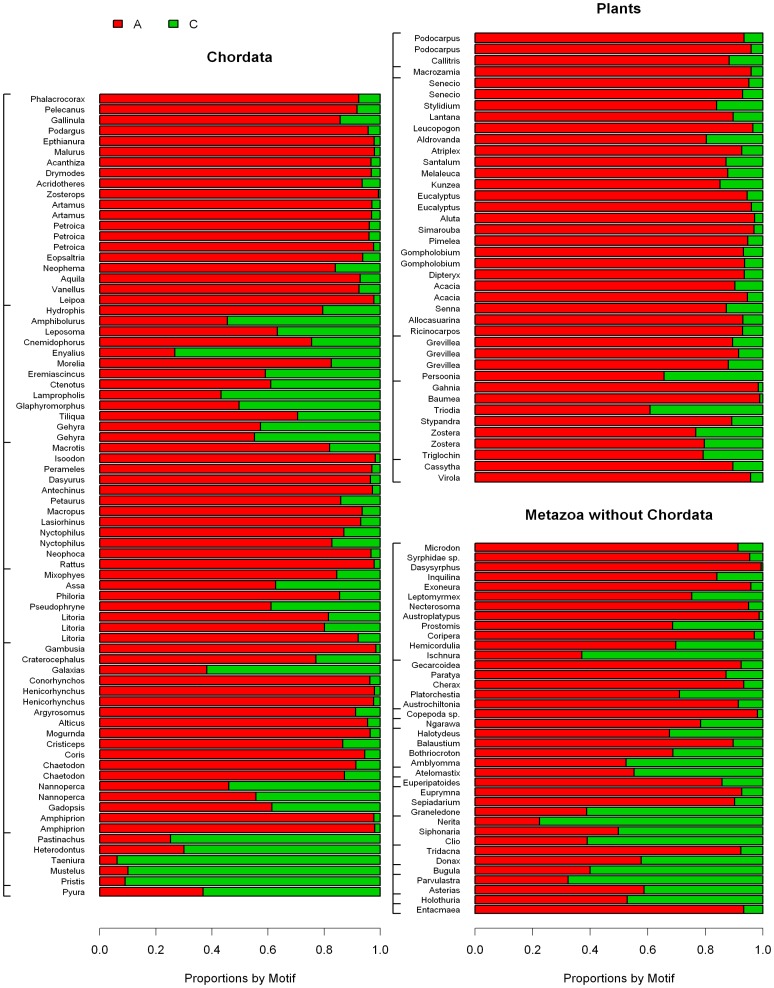
Proportion of A/T and C/G homopolymers for each species. (A) Chordates, (B) Animals without Chordata (C) Plants; species follow the same order as in Dataset S1 and in Figures 1 and 2.

**Figure 8:**
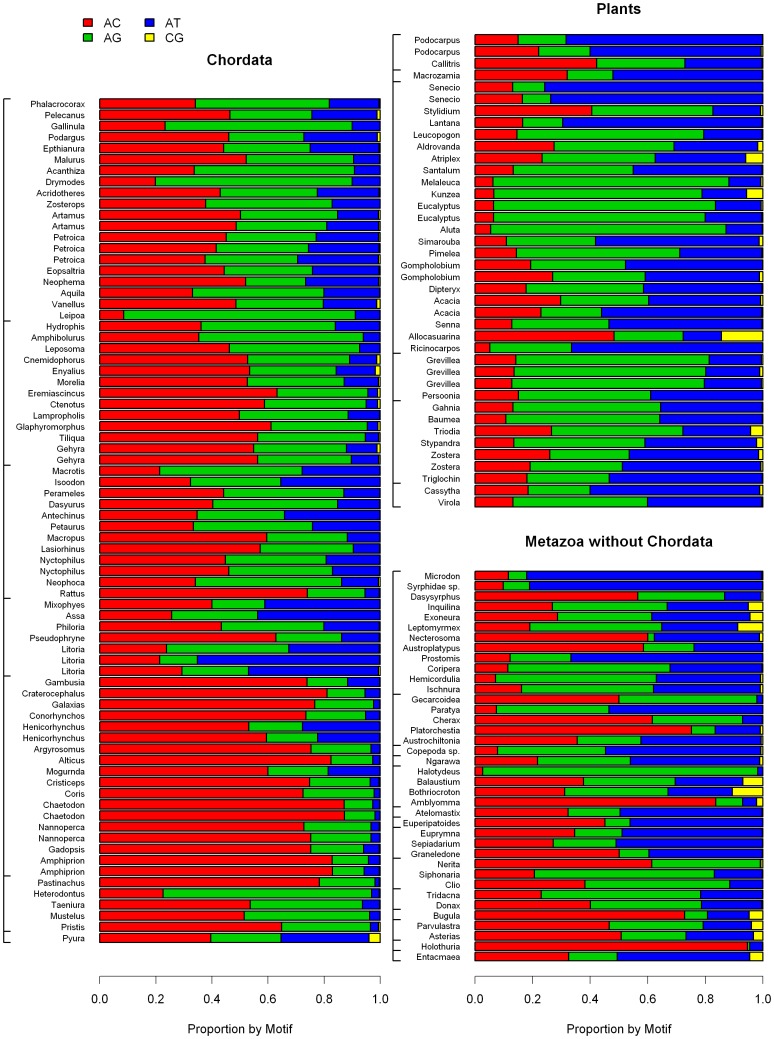
Proportion of all four dinucleotide motifs within the total dinucleotide microsatellite coverage for each species. (A) Chordates, (B) Animals without Chordata (C) Plants; species follow the same order as in Dataset S1 and in Figures 1 and 2.

**Figure 9:**
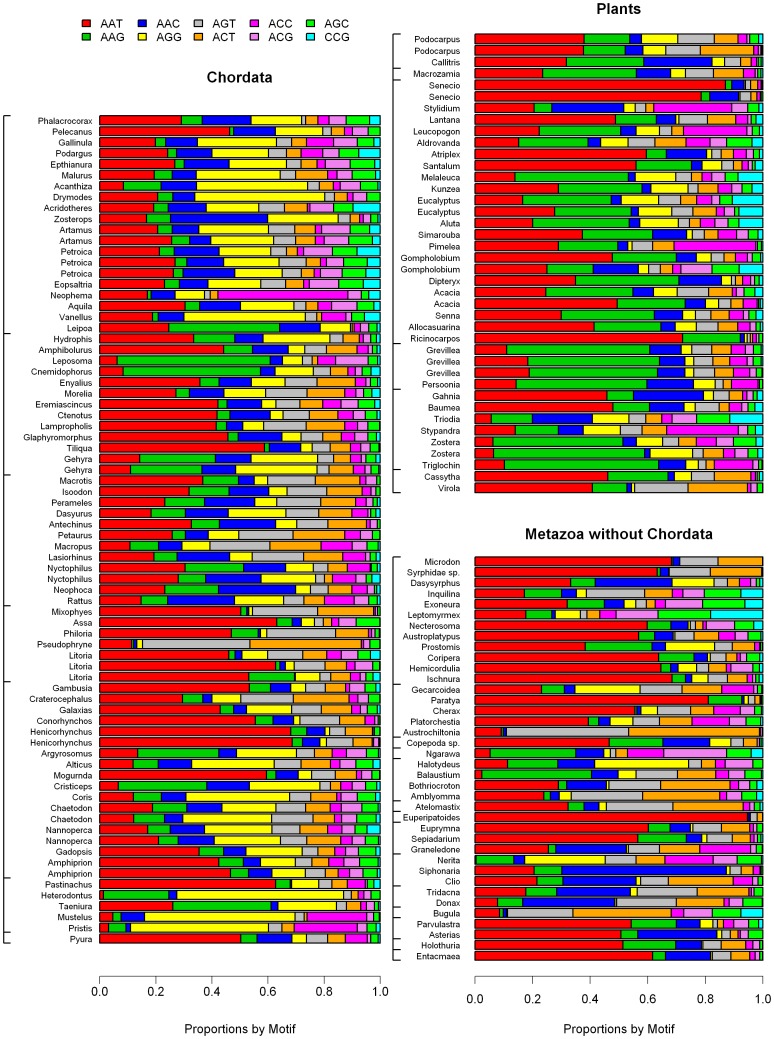
Proportion of all ten trinucleotide motifs within the total trinucleotide microsatellite coverage for each species. (A) Chordates, (B) Animals without Chordata (C) Plants; species follow the same order as in Dataset S1 and in Figures 1 and 2.

The proportion of each motif was expressed as the coverage of the motif divided by the coverage of all microsatellites of the same motif length with barplots of proportions of the most frequent motifs in Figures 7, 8, 9 and S2, S3, S4.

PolyA was the far most frequent homopolymer in the dataset, but the proportion of polyC was typically high in the studied Chondrichthyes, Squamata and Echinodermata (Figure 7, Dataset S1). Spearman rank correlation test indicated a significantly negative correlation between the proportion of PolyA/T within homopolymers and GC % of the 454 sequences (ρ = -0.349, P<1e-05, n = 154; Figure S2A). However, the maximum GC% in the dataset was 48%, thus GC% alone cannot explain polyC proportions as high as 94%.

Among dinucleotide motifs, CG was clearly the rarest and in most of the species its proportion was close to 0 (Figure 8). Few general tendencies could be drawn for the proportions of the remaining three dinucleotide motifs. AT motif proportions were negatively correlated to GC% of the 454 sequences (Spearman rank correlation test; ρ = −0.625, P<2.2e−16, n = 154, Figure S2B), but no clear phylogenetic pattern was observed. The motif AC was the most frequent in most of the chordates, especially in Actinopterigii, while it was the second rarest (after the CG motif) in plants. Again no clear pattern was observed in the remaining species.

Among the ten trinucleotide motifs, AAT was the most frequent. The AAT proportion was higher than 0.1 in 138 species, while this number is only 72 for AAG and 62 for AAC. AAG motifs are predominant in several plant species. No other tendency was detected (Figure 9). Among tetranucleotide motifs, AAAT was the most frequent in plants, with no clear phylogenetic pattern (Figure S3).

The number of penta- and hexanucleotide motifs were relatively low, thus it was difficult to provide a good estimation of their proportions from our data. No pattern appeared in the relative frequencies of the motifs (Figures S4–S5). However, it is interesting to note that some motifs were never present in the entire dataset: AAAGCT, AACCGC, AACGCG, AAGCGC, AAGCTT, ACCCGC, ACCGCG, ACCGGT, ACGCCT, ACGCGG, ACGCGT, ACGCTG, ACGGGT, ACGTCG, AGAGCT, AGATCG, AGCCGT, AGCGAT, AGCGCC, AGCGCG, AGCGCT, AGCTCG, AGGCGT, CCCGGG, CCGCGG. Although these motifs are generally rich in CG, there does not appear be any other obvious links among them.

## Discussion

Despite a considerable body of work on their evolution, there is no strict consensus on the definition of a microsatellite [7], [34]. While they are generally defined as tandem repetitions of short motifs, there is no standard cut off limit for the minimum length of microsatellites. Mutability studies indicated that for mono and dinucleotide repetitions the slippage rate changes around 10 bases, thus this length could serve as a minimum cut off [35]. However, Leclercq et al. [36] have found that rates of tandem insertions and deletions increased exponentially with microsatellite size, but they did not detect lower threshold length for slippage. Whilst many studies use a minimum number of base pairs [23], [26], [31], others use the minimum number of repetitions [20]–[22], [27] and both criteria vary between studies. Furthermore, the degree of the degeneracy allowed also differs among search algorithms [37]–[41]. As a consequence, direct comparison of the results of different studies is problematic and it is important to compare distantly related species with the same method as we have done in this study. Previous studies of microsatellite distribution of distantly related species are limited to model species with only few species representing a phylum [19], [26], [27], [30]. Additionally, studies focusing within a single phylogenetic group are also often limited to a small number of species with assembled genomes or whole genome shotgun data [20], [21], [23], which makes testing if any phylogenetic pattern is mirrored in the microsatellite distribution impossible. Can the results obtained for one phylogenetic group from a small number of species be generalized for the whole group? Can the pattern observed between groups be generalized?

In this study we used 6–125 Mb of shotgun data from each of the 154 species studied. Our phylogenetic sampling is biased: almost half of the species are Chordata, and the plants were only represented by seed plants (Spermatophyta) and most of them were flowering plants (Magnoliophyta). The dataset included little studied phyla such as Cnidaria, Bryozoa, Echinodermata and Onychophora but we had only a few species representing each one of them. However, to our knowledge, this is the first study describing microsatellite distribution of such a large number of non-model species, representing several distantly related phyla, with a good sampling of vertebrates, flowering plants, several species of arthropods, molluscs and some species representing minor phyla. Furthermore, apart from the number of beads loaded, the protocol of sequencing was the same for all species. This considerably reduces the heterogeneity due to technical biases.

### Strong Heterogeneity of Microsatellite Coverage

The most striking outcome of our results is the extreme heterogeneity of microsatellite coverage and composition of different phylogenetic groups older than ca. 200 Mya. Despite this large heterogeneity, some clear trends emerged: (i) Seed plants (Spermatophyta) have lower microsatellite coverage than animals in general and relatively low heterogeneity in microsatellite coverage; (ii) a phylogenetic pattern is clearly observable within chordates; (iii) Strong heterogeneity was observed within and among non-chordate metazoan phyla with a weak indication of phylogenetic patterns for Arthropods. Although these findings appear contradictory, analyzing these data in light of divergence times, resolves this apparent ambiguity.

The relatively low variability among microsatellite coverage among different flowering plant species can be explained by a recent radiation of flowering plants. The MRCA of all studied Magnoliophyta is younger than 200 Mya. This divergence time was apparently not long enough to lead to strong differentiation of microsatellite coverage of different species. In Chordata, a positive autocorrelation of clades younger than 200 Mya, also indicated the absence of strong heterogeneity within these clades. The MRCA of the studied vertebrates dates to 400–500 Mya, and the separation of different vertebrate classes dates back to 200–400 Mya, leading to differentiation of microsatellite coverage between them and an apparent phylogenetic pattern. It thus appears that we detected a phylogenetically conserved signal, since in this phylum the independent evolution of the lineages has not been in operation long enough to totally scramble the microsatellite pattern of the vertebrate ancestor, but was sufficient to produce notable differences. Our sampling of vertebrate classes was deep, whilst in other animal phyla we probably could not detect a phylogenetic pattern as our species coverage was inadequate to obtain statistical significance. Although divergence times amongst different orders in Arthropoda were within the timeframe of 200 Mya where a positive correlation was still detected in vertebrates, the species representation within each order was small. For example, although we had 24 species of Arthropoda, the most represented order (Coleoptera) had only four species and from most orders we had only 1 or 2 species, strongly reducing the power of statistical testing. However, in spite of the problems due to the low species coverage, it is clear that a very strong heterogeneity of microsatellite coverage within phyla and within classes of invertebrates exists. The MRCA of the different phyla or different classes of invertebrates dates back to 400–900 Mya. This time appears to be long enough to erode the microsatellite pattern of their common ancestor.

Our study highlights three important points. Firstly, patterns of microsatellites composition within phylogenetic groups are broken down by time. Galindo *et al.* [19] found by analyses of microarray hybridization, that total microsatellite content reflects the phylogeny of the primates studied. This could be observed, since primates form a recent clade. However, in their analyses, the rest of the Eukaryotes were represented only by five vertebrates (including four mammals), a *Drosophila* and two plant species, and thus generalization was impossible. Our study highlights this point. Although microsatellite coverage of closely related species tends to be similar, this relationship breaks down with increasing evolutionary distance between species.

Secondly, apparent patterns that may arise with limited sampling are likely to be shown to be false with greater sampling due to a very strong variation among a large number of species. For example, while analyzing all four available genome sequences of Hymenoptera, Pannebakker *et al.* [21] found that all of them had higher microsatellite coverage than any of the eight other Arthropod species they used for comparisons. This is not the case in our study, as the three non-model Hymenoptera species had comparable microsatellite coverage ranges to the other insect orders, and much lower coverage than Decapodes, an order not sampled by Pannebakker *et al.* [21]. Thus, by increasing the number of species studied for each phylogenetic group, considerable heterogeneity is observed in microsatellite composition and coverage. This is a very important take home message from this study.

Lastly, quantification of microsatellite coverage/abundance/amount within a clade (especially if older than ca. 200 Mya) does not make much sense in light of the observed variability. Lagercrantz *et al*. [42] have found that plants have about 5 times less microsatellite DNA than mammals. Although this conclusion is possible if pooling data from database searches, as was done in Lagercrantz *et al.*’s paper, this approach does not take into consideration the within group variability. Our data also suggests that microsatellite coverage is lower in plants than in mammals, but in the light of the between species variation quantifying microsatellite coverage differences between groups of species are meaningless. Although, we concluded that plants have lower microsatellite coverage than animals in general, it is also clear that some animals, *e.g.* birds, have comparable levels to that of plants.

### Repeat Type

Two major mechanism have been proposed to explain the formation of microsatellites (reviewed in [6]): spontaneous formation from unique sequences by substitution or insertion [8], [43] creating proto-microsatellites, then elongation, or spread of proto-or full microsatellites by transposable elements [10]. We hypothesize that the formation of proto-microsatellites is less likely for longer motifs than for shorter ones, which would explain why dinucleotide motifs are the most frequent in the majority of the taxa, and why penta and hexa microsatellites are rare.

Describing the most frequent motifs is a basic analysis in microsatellite distribution papers. What is the most frequent motif for a single species is very clear, but as the number of species increases in the studies the relative frequencies of the motifs can vary considerably. From our analyses, very few clear trends can be drawn. A/T homopolymers are more frequent than C/G homopolymers in most species studied. However, all studied Chondrichthya and Squamata are rich in polyC as are some other species poorly studied for microsatellite coverage from Echinodermata, Mollusca and Bryozoa, suggesting that polyCs are not as rare among Eukaryotes as it is suggested in the literature [23], [24], [27], [28], [31] (but see [20]). Since most genome have a CG content between 35%–45% (Dataset S1), CG percentage cannot be the only explanation of the generally high polyA proportion. Tóth *et al.* [31] suggested that the polyA tails of retroposed sequences such as LINEs, and processed pseudogenes are responsible for the higher proportion of A/T rich microsatellites. Although this is plausible explanation, it is necessarily a partial one. Avian LINE elements do not have polyA tails, yet the proportion of polyA homopolymers in birds is as high as in mammals [44].

CG dinucleotide microsatellites are clearly rare in all genomes studied both in this study and in the literature [20], [23], [24], [27], [28], [31]. This cannot be explained by low C/G content of the genome, or insufficient sampling, thus it looks like a genuine pattern. CpG dinucleotides not situated in CpG islands can undergo methylation of cytosine in most Eukaryote genomes [45]. Methylated cytosine tends to mutate to thymine, which can be an explanation for the underrepresentation of CpG dinucleotides in genomes [46], [47] and consequently the low coverage of microsatellites with CG motif. [47].

For longer motif classes, increasingly there are a larger number of possible base pair combinations and there is a much greater variability in what motif is the most frequent in each dataset. Therefore there is a difficulty in detecting any consistent pattern. There have been several hypotheses put forward to explain the apparent abundance or lack of certain motif types in previous studies. For example generally high frequency of AT motifs in fungal genomes is suggested to be the consequence of high A/T content of the genomes and the relative ease of strand separation compared with C/G tracts [23], [48]. The high abundance of GT repeats in mammals has been linked to formation of Z-DNA [49] and regulation of gene expression [50]. The high proportion of A/T rich microsatellite motifs, particularly the A(2–5)N motifs is attributed to mutations that appear in the polyA tail of retro transposed elements [31]. In light of the daunting variability of the microsatellites coverage and composition of different Eukaryotes, it is unlikely that microsatellite composition is shaped by only a few universal forces. Factors such as mutation mechanisms, microsatellite type (allele length, repeat unit length, composition), genomic context, and selection are all factors influencing microsatellite composition of species [6]. As a result, the pattern of the microsatellite composition from a common ancestor of a clade breaks down rapidly after divergence. Furthermore, we think that it is likely that microsatellite composition is driven by chance events as well such as a spread of different transposable elements. Reports of association between microsatellites and transposable elements suggest that at least some microsatellites are spread via mobile elements either as mature or proto-microsatellites [51]–[56]. This could explain different microsatellite composition between closely related species, if they are dominated by different transposons [57]. However, systematic genome scale studies are rare, and this is likely to be due to the consequence that transposable element detection is difficult when based on low coverage genomic data, especially with small fragment sizes.

### Conclusions

Our results reveal a very strong heterogeneity of microsatellite composition within all clades older than ca. 200 Mya. This finding clearly indicates that data from model species does not reflect the inherent variability of Eukaryotes, and thus conclusions drawn from a limited number of species should be treated with care. Although, it is likely that recent phylogenetic lineages show a consistent pattern in their microsatellite composition (as it was shown within vertebrates), a thorough sampling within these groups would be necessary to reveal this pattern. While our sampling was acceptable for vertebrate species, this phylum represents only a fraction of the Eukaryotes. Sampling of the rest of the Eukaryotes was insufficient to reveal a phylogenetic pattern, but even with limited information, we could clearly point out that generalizing information of microsatellite content from few species to a whole group can only be justified if they are from a very recent clade.

## Materials and Methods

### DNA Sequencing and Species List

The sequences used in this study were obtained from collaborative microsatellite development coordinated by one of us (MGG). Therefore, the species were chosen by several independent research groups based on their own research interest and thus the species were not selected to obtain a comprehensive phylogenetic coverage of the Eukaryotes. Dataset S1 lists the 154 species examined within this study and includes information on the taxonomy of each species and the contact person, as well as the total length of the 454 shotgun sequences, and the number of bases in microsatellites in each motif type. Sequences have been deposited to the Dryad database (http://dx.doi.org/10.5061/dryad.f1cb2, http://dx.doi.org/10.5061/dryad.jd183). Taxonomical divisions are in agreement with the NCBI’s Taxonomy database wherever available. Following Gardner *et al*. [58], DNA from all species was sheared by Covaris™ and 500 ng of purified DNA was used for 454 FLX Titanium library (Roche Applied Science) preparation, according to the manufacturer’s protocols using parallel sample cleanup and RL MID adapters. Emulsion PCR (emPCR) was carried out at a ratio of three copies per bead. Each Titanium PicoTiter plate contained two gaskets and two million beads were loaded in each half which was the equimolar pooling of libraries from 2–4 species in each of them. Sequencing was done with 200 cycles. Sample preparation and analytical processing, such as base calling, were performed at Australian Genome Research Facility Ltd (AGRF, Brisbane Australia), according to the manufacturer’s protocol for the Titanium series.

### Divergence Times and Phylogeny

Phylogenetic relationships and divergence times for the plant species were estimated using the online software Phylomatic [59] and the Branch Length Adjuster (BLADJ) algorithm in the Phylocom 4.2 software [60]. Phylomatic matches taxon sample names with information on seed plant phylogeny according to Angiosperm Phylogeny Group III [61] (source tree R20100701) to derive evolutionary relationships among samples. The BLADJ algorithm constrains the age of nodes included in the sample according to the dated molecular phylogeny of Wikström *et al*. [62], and for nodes where an age estimate is unavailable, sets the age as the midpoint between constrained nodes to produce an ultrametric topology. For the animal tree, relationships among phyla are as per the recent study of Dunn *et al*. [63], and were supplemented with information from finer scale studies including Arthropoda [64], Mollusca [65], Mammalia [66] and Squamata [67]. The animal tree was input into Phylocom and made ultrametric using minimum age constraints according to Benton and Donoghue [68] as reported on the Date-a-Clade website (http://www.fossilrecord.net/dateaclade) and the BLADJ algorithm. The resulting phylogenetic trees (Figures 1 and 2) were drawn with the APE package [69] within the R language [70].

### Data Analyses

All sequences that passed the standard quality filtering of 454 platforms were searched for perfect microsatellite tracks with custom Perl scripts (available from the corresponding author). The minimum number of repetitions for inclusion was twelve for homopolymer tracks and five for di-hexa-nucleotide motif classes. Microsatellites were classified according to (i) motif sequence, (ii) repeat unit length (mono-hexa). We adopted the alphabetical minimal names for motifs with circular permutation and reverse complementary sequences grouped together (*e.g.* AAC for AAC, ACA, CAA, TTG, TGT, and GTT). Microsatellite coverage was given by the total number of bases of microsatellites in one Mb of sequences. For describing the most frequent motifs within each motif length, the coverage values of each motif was transformed into a proportion by dividing them by the total coverage of the microsatellites of a given repeat unit length.

Microsatellite coverages of different phylogenetic groups were compared by two sample Wilcoxon test using R [70]. Autocorrelation of microsatellite coverage within clades was assessed, after log transformation, with correlograms on Moran’s autocorrelation index *I*, computed by the APE package [69] within the R language [70]. Species were pooled for comparison in the largest possible clades where the MRCA were younger than the following cut off limits: 50 Mya and then in multiples of 100 Mya.

## Supporting Information

Figure S1 **Proportion of microsatellites of each repeat unit length.** Coverage of microsatellites of each repeat unit length and homopolymers is divided by the total microsatellite and homopolymer coverage; species follow the same order as in Dataset S1 and in Figures 1 and 2.
(TIF)

Figure S2 **Correlation between GC% of sequences and A/T rich microsatellite proportions.**
(TIF)

Figure S3 **Proportion of the six most frequent tetranucleotide motifs within the total tetranucleotide microsatellite coverage.** Species follow the same order as in Dataset S1 and in Figures 1 and 2.
(TIF)

Figure S4 **Proportion of the six most frequent pentanucleotide motifs within the total pentanucleotide microsatellite coverage.** Species follow the same order as in Dataset S1 and in Figures 1 and 2.
(TIF)

Figure S5 **Proportion of the six most frequent hexanucleotide motifs within the total hexanucleotide microsatellite coverage.** Species follow the same order as in Dataset S1 and in Figures 1 and 2.
(TIF)

Dataset S1 Species list and the number of bases in microsatellites or homopolymers for each investigated species. Columns included are: Species names, taxonomic groups, contact person, total length of the 454 sequences, GC proportions of 454 dataset, and the number of base pairs of microsatellites for each repeat unit length and for each motif.
(CSV)
