# Correction: Breakdown of Phylogenetic Signal: A Survey of Microsatellite Densities in 454 Shotgun Sequences from 154 Non Model Eukaryote Species

**DOI:** 10.1371/annotation/929133fb-96cd-4223-a8f4-ff8c75c6fd5f

**Published:** 2013-12-17

**Authors:** Emese Meglécz, Gabriel Nève, Ed Biffin, Michael G. Gardner

An error occurred in the script of the analysis pipeline that modified figures 9, S3, S4, S5 and the supplementary dataset S1 of this article.

Please refer to the corrected information below.

Figure 9: 

**Figure:**
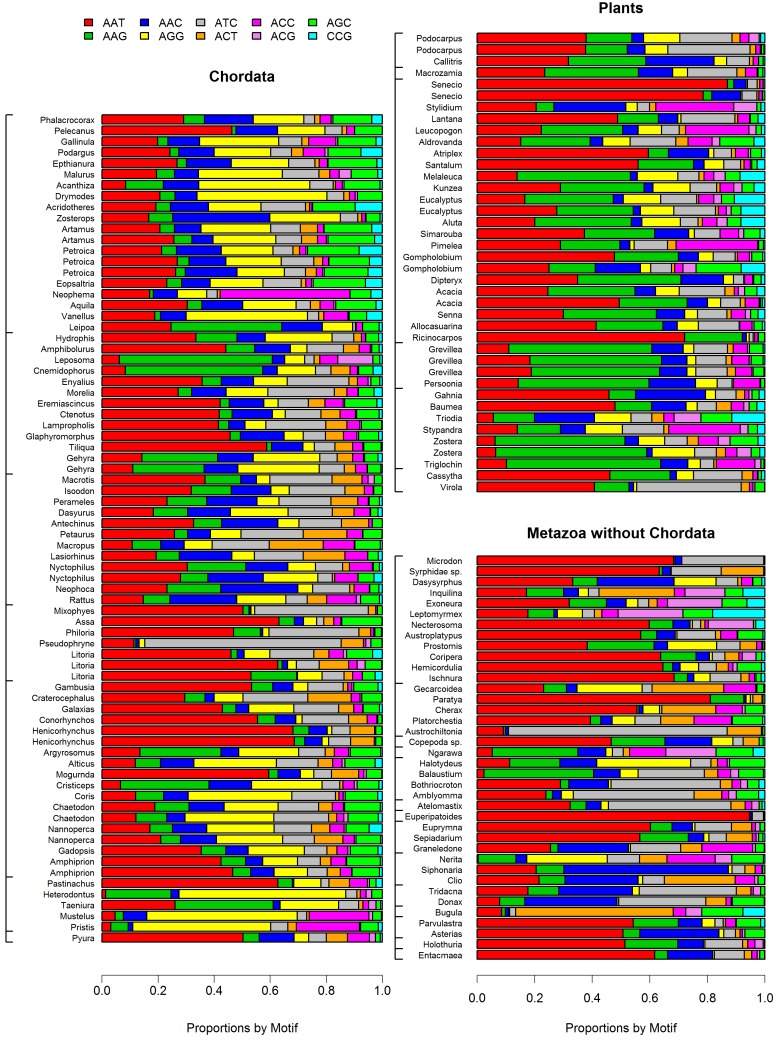


Figure S3: 

Figure S4: 

Figure S5: 

Dataset S1: 
