# Supplementary figures and images for: Breakdown of Phylogenetic Signal: A Survey of Microsatellite Densities in 454 Shotgun Sequences from 154 Non Model Eukaryote Species
Source: PLoS One. 2012 Jul 16;7(7):e40861. doi: 10.1371/journal.pone.0040861 (PMC3397955; doi:10.1371/journal.pone.0040861)

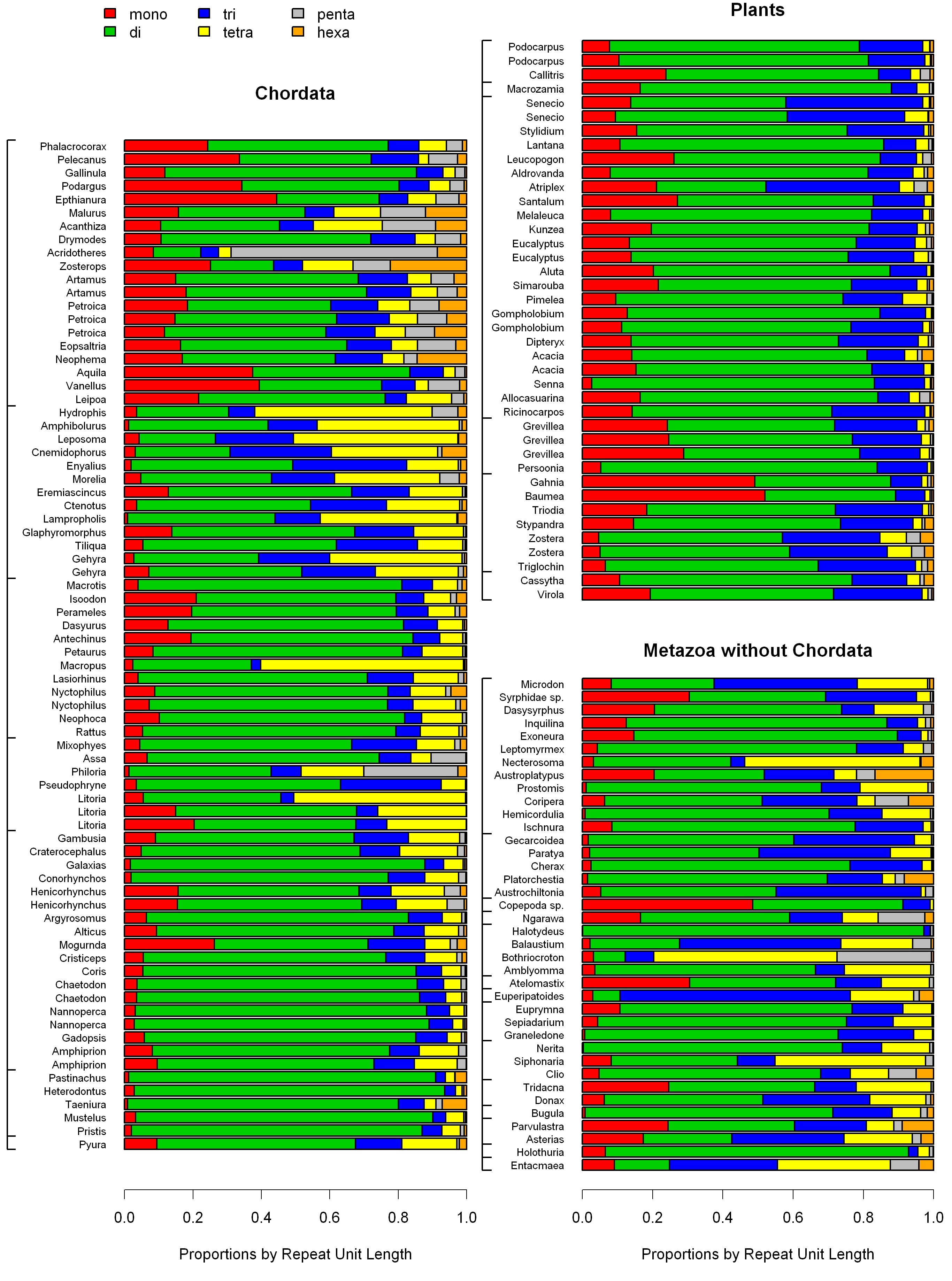

Supplement: Figure S1 — Proportion of microsatellites of each repeat unit length. Coverage of microsatellites of each repeat unit length and homopolymers is divided by the total microsatellite and homopolymer coverage; species follow the same order as in Dataset S1 and in Figures 1 and 2. (TIF) [file pone.0040861.s001.tif]

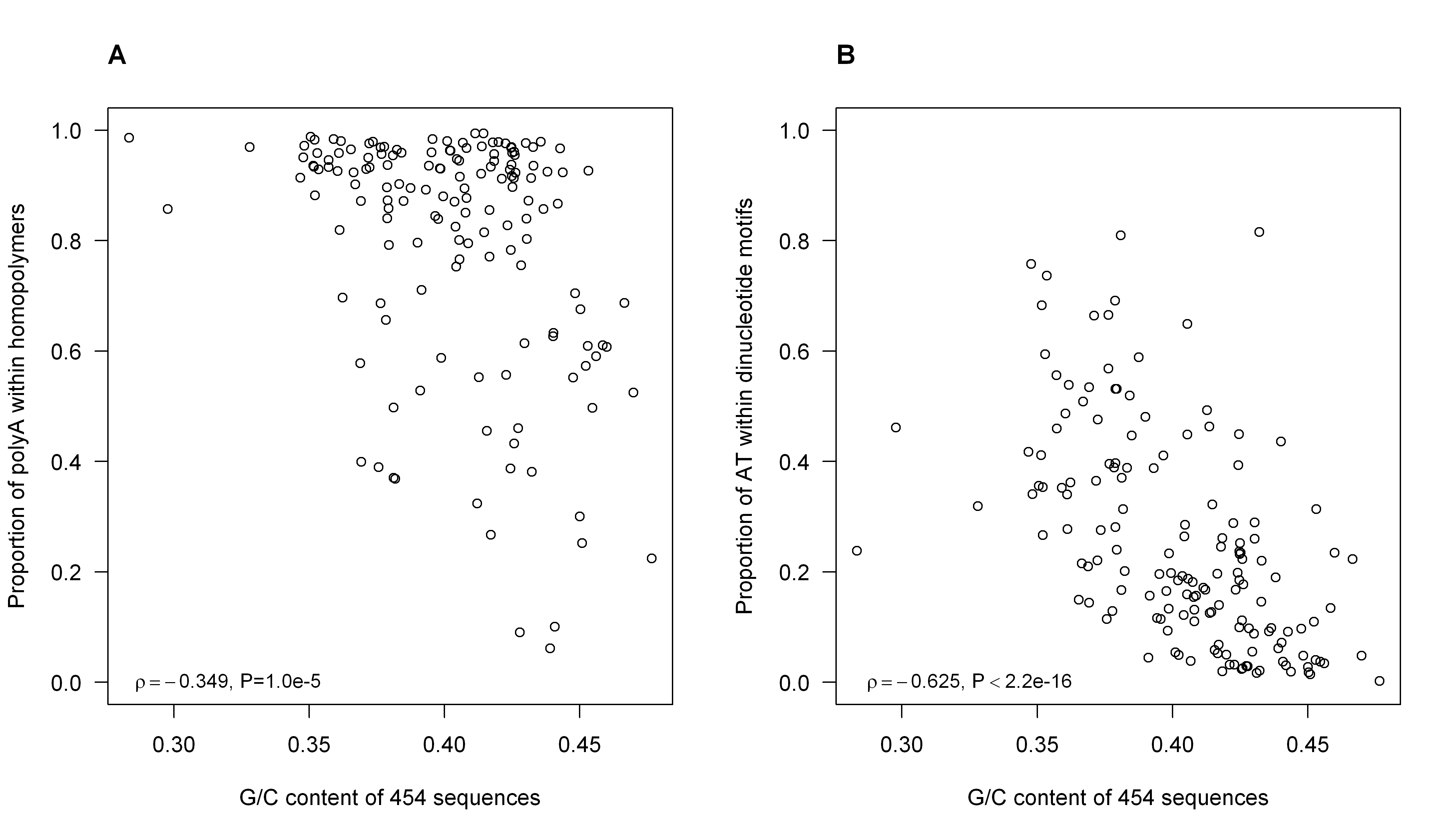

Supplement: Figure S2 — Correlation between GC% of sequences and A/T rich microsatellite proportions. (TIF) [file pone.0040861.s002.tif]

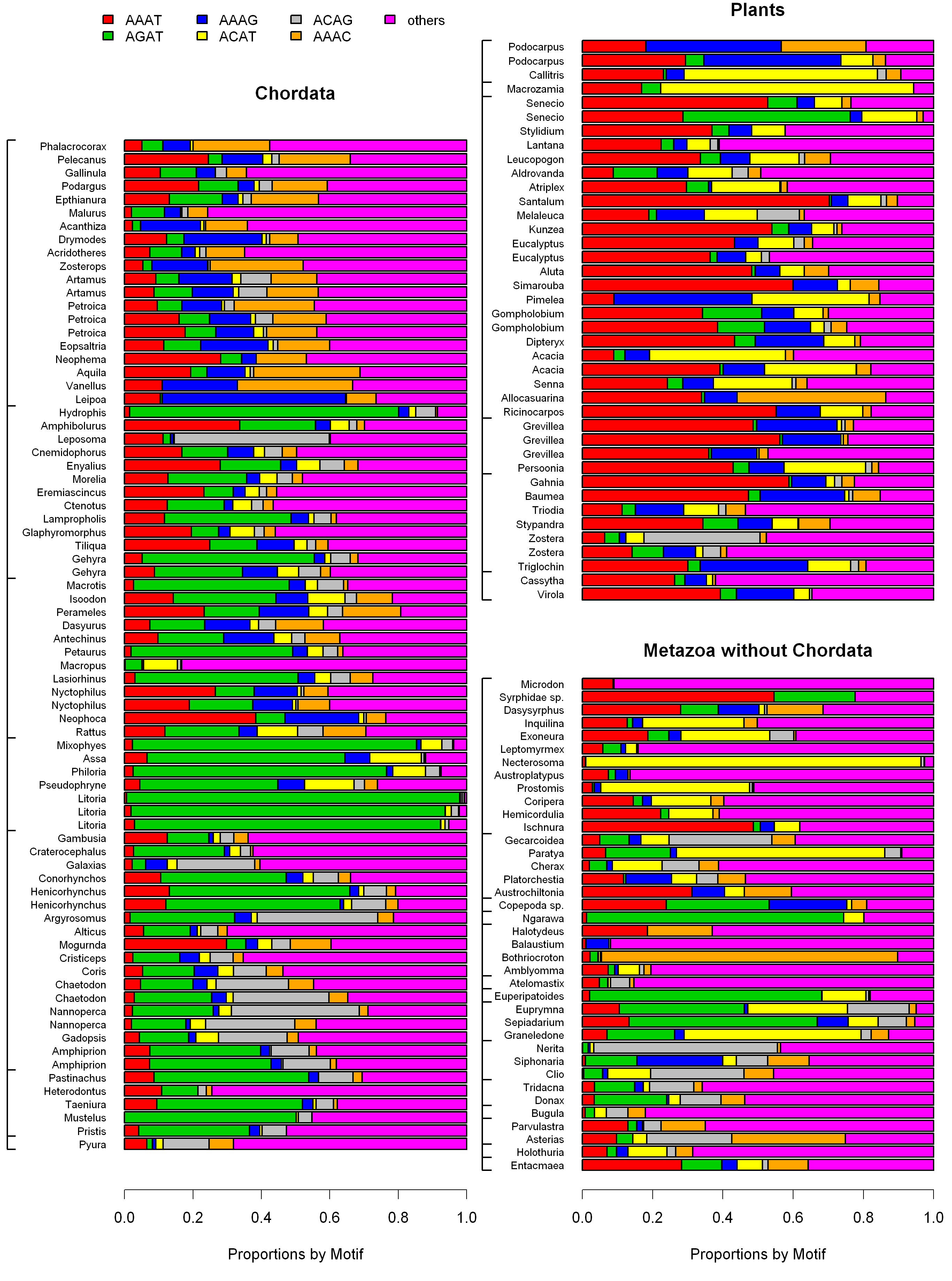

Supplement: Figure S3 — Proportion of the six most frequent tetranucleotide motifs within the total tetranucleotide microsatellite coverage. Species follow the same order as in Dataset S1 and in Figures 1 and 2. (TIF) [file pone.0040861.s003.tif]

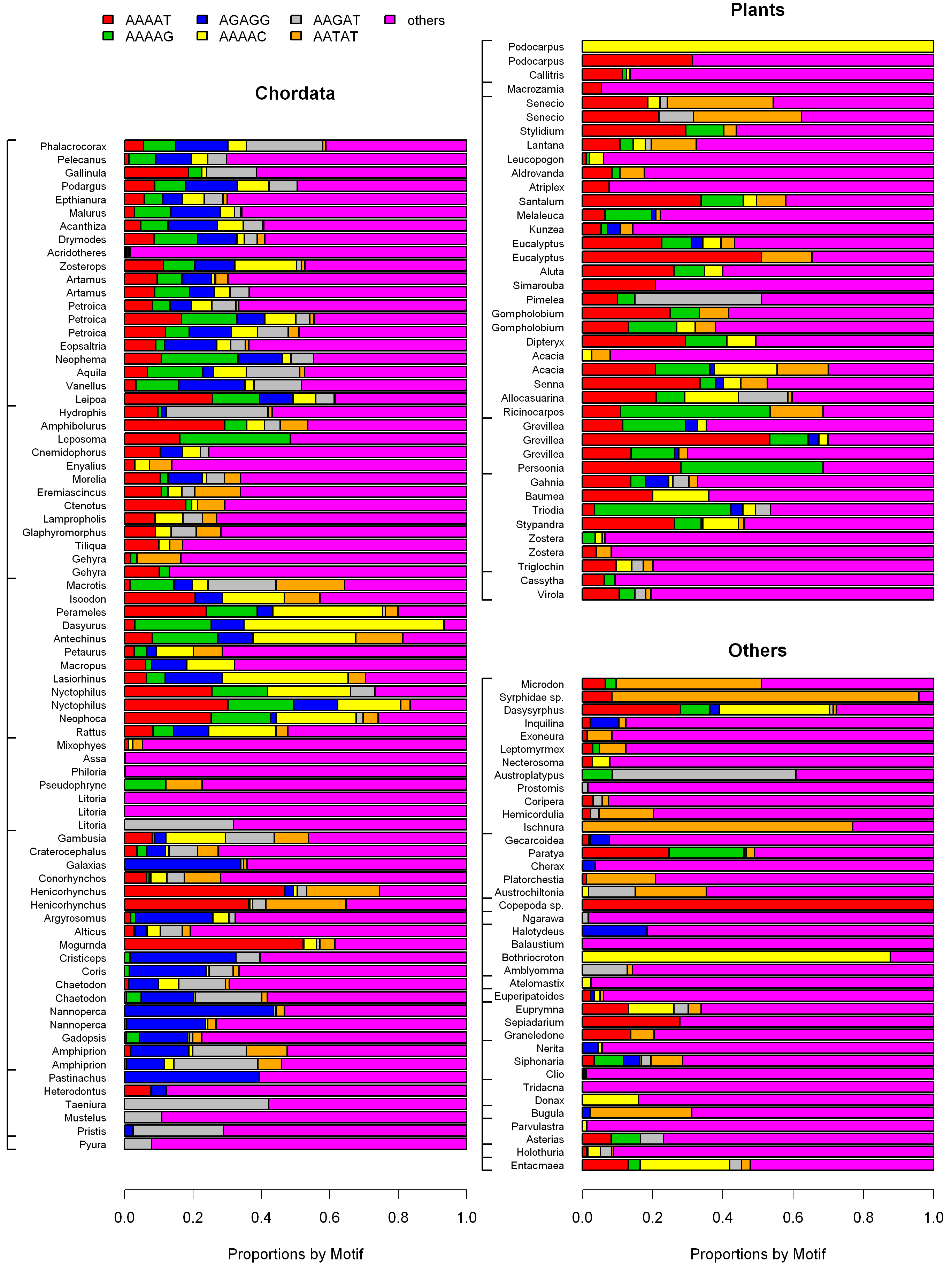

Supplement: Figure S4 — Proportion of the six most frequent pentanucleotide motifs within the total pentanucleotide microsatellite coverage. Species follow the same order as in Dataset S1 and in Figures 1 and 2. (TIF) [file pone.0040861.s004.tif]

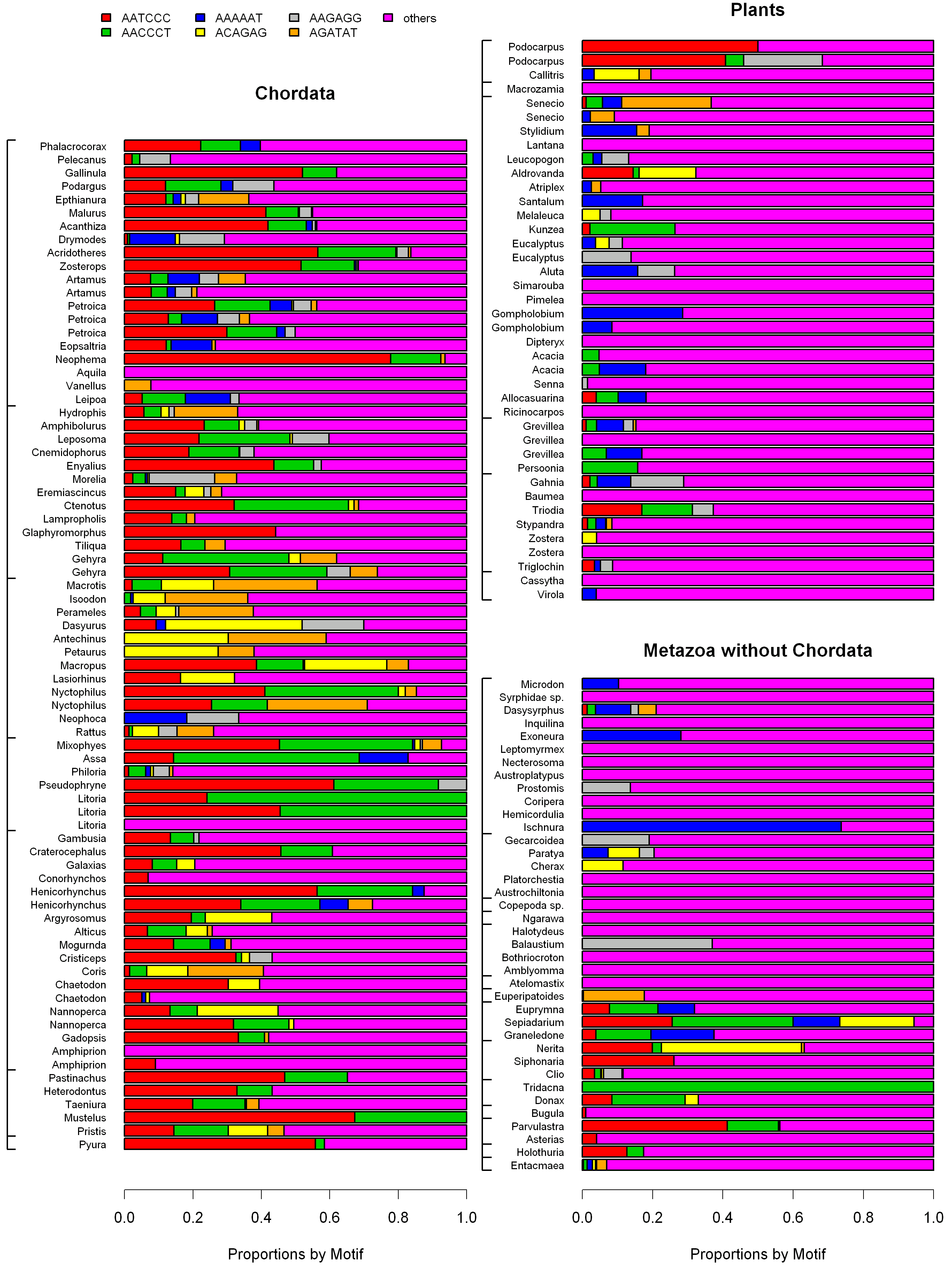

Supplement: Figure S5 — Proportion of the six most frequent hexanucleotide motifs within the total hexanucleotide microsatellite coverage. Species follow the same order as in Dataset S1 and in Figures 1 and 2. (TIF) [file pone.0040861.s005.tif]
