# Supplementary figures and images for: Correction: Breakdown of Phylogenetic Signal: A Survey of Microsatellite Densities in 454 Shotgun Sequences from 154 Non Model Eukaryote Species
Source: PLoS One. 2013 Dec 17;8(12):10.1371/annotation/929133fb-96cd-4223-a8f4-ff8c75c6fd5f. doi: 10.1371/annotation/929133fb-96cd-4223-a8f4-ff8c75c6fd5f (PMC3866329; doi:10.1371/annotation/929133fb-96cd-4223-a8f4-ff8c75c6fd5f)

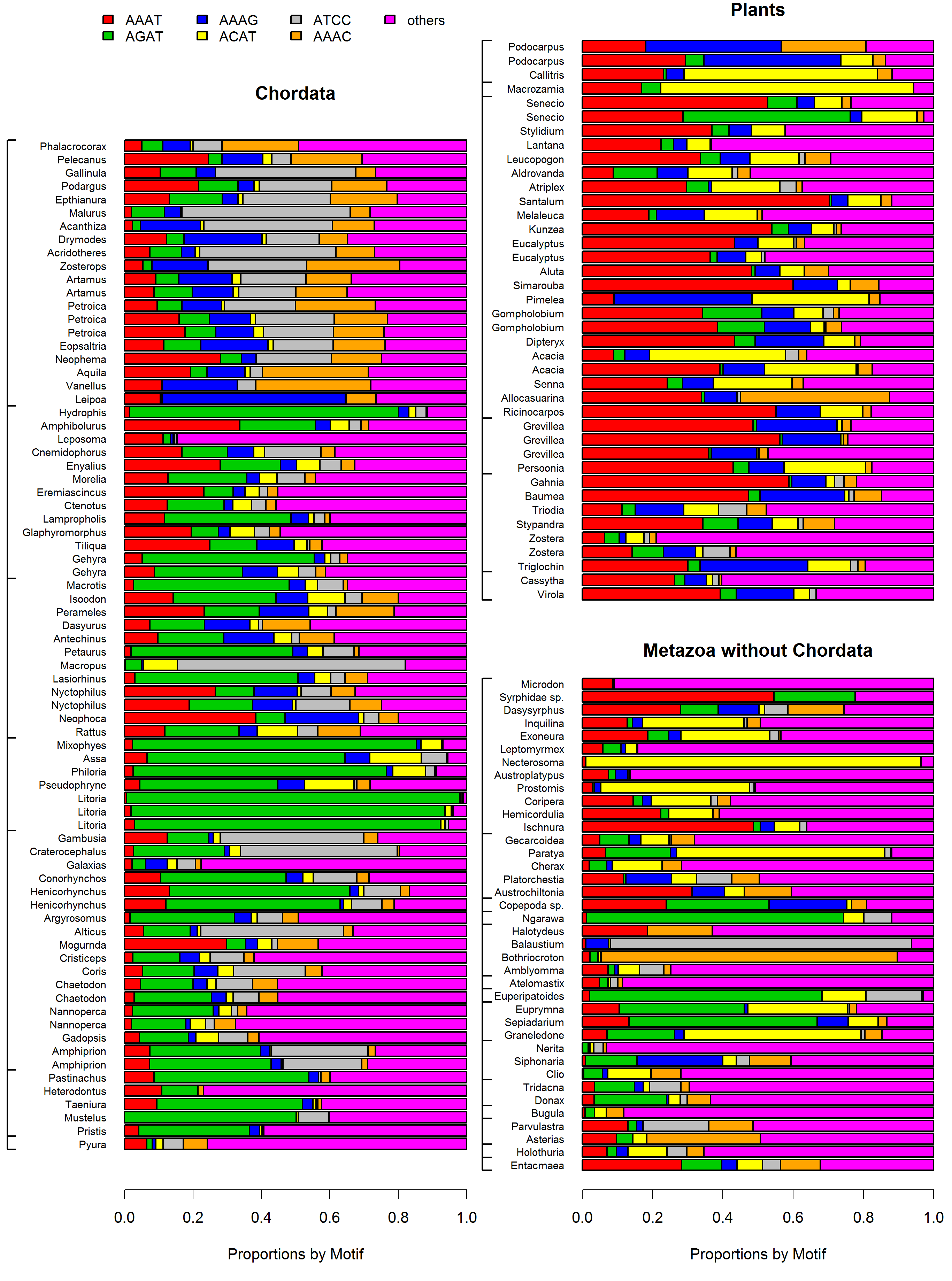

Supplement: Supplementary file 1 [file pone.929133fb-96cd-4223-a8f4-ff8c75c6fd5f.s001.tif]

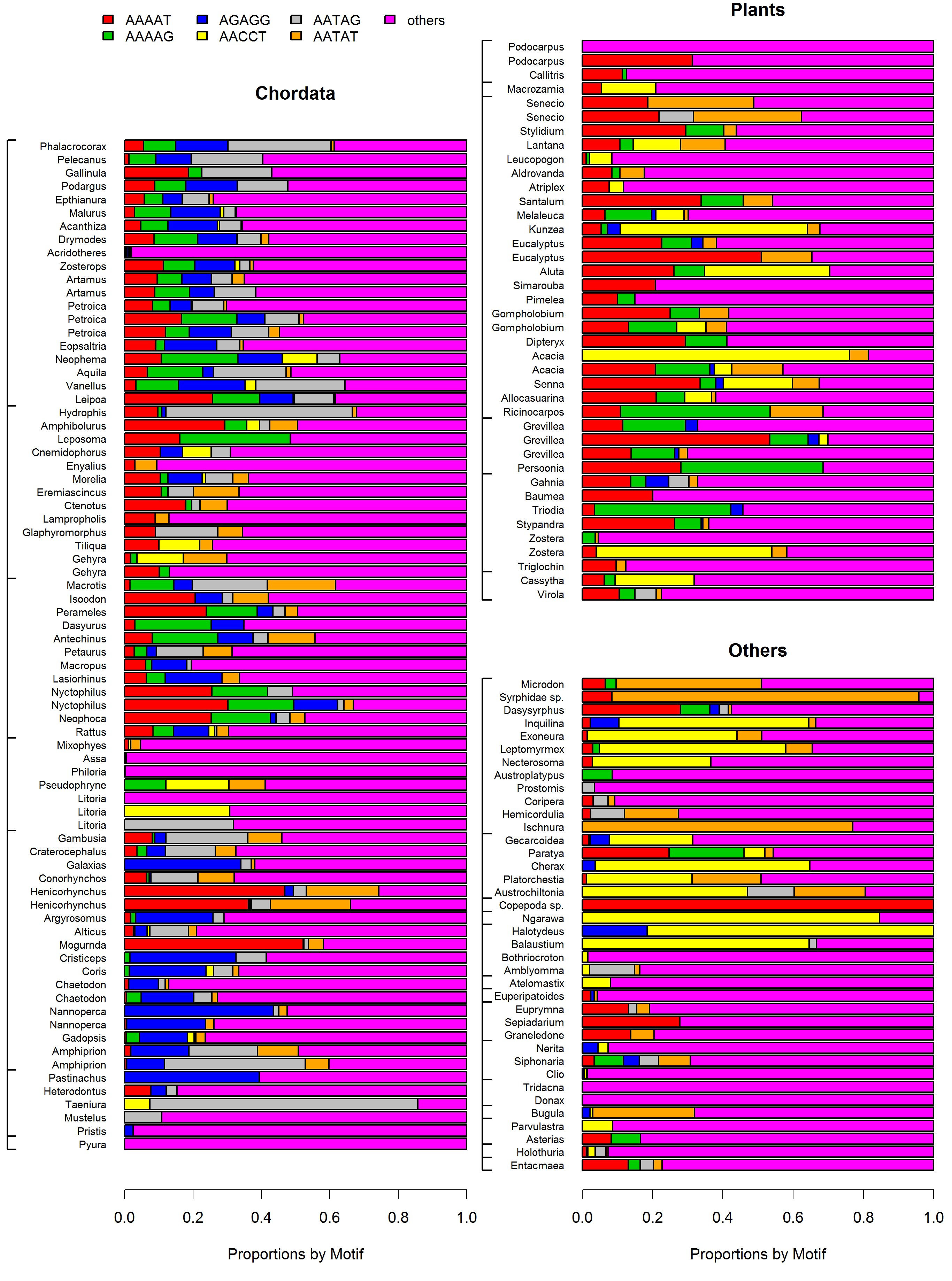

Supplement: Supplementary file 2 [file pone.929133fb-96cd-4223-a8f4-ff8c75c6fd5f.s002.tif]

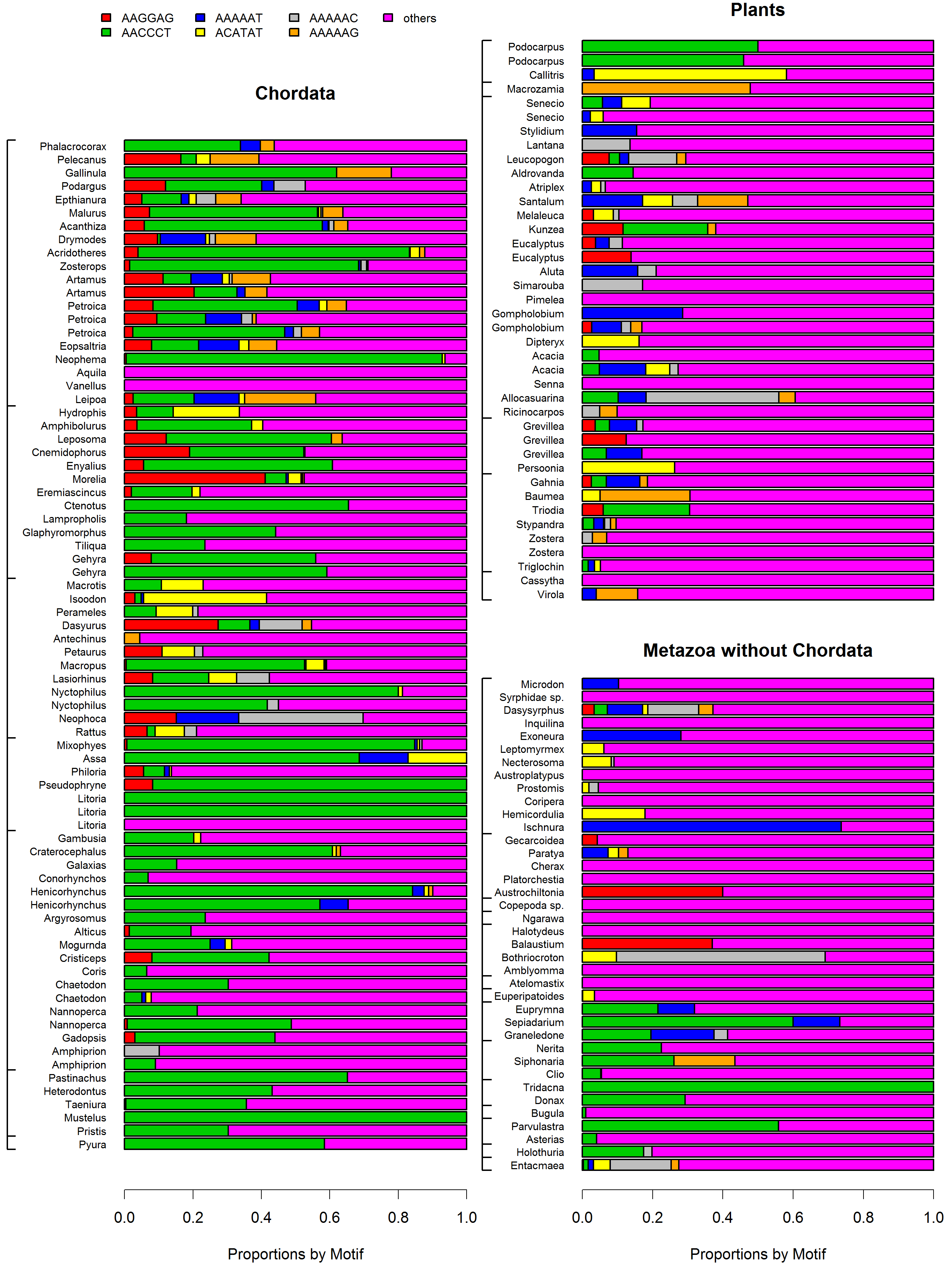

Supplement: Supplementary file 3 [file pone.929133fb-96cd-4223-a8f4-ff8c75c6fd5f.s003.tif]
